# Social support: mediating the emotional intelligence-academic stress link

**DOI:** 10.3389/fpsyg.2023.1218636

**Published:** 2023-09-05

**Authors:** Muhammad Shariat Ullah, Sharmeen Akhter, Muhammad Abdul Aziz, Muhaiminul Islam

**Affiliations:** Department of Organization Strategy and Leadership, Faculty of Business Studies, University of Dhaka, Dhaka, Bangladesh

**Keywords:** emotional intelligence, academic stress, social support, structural equation modeling, PLS, tertiary level students

## Abstract

**Introduction:**

This study examines the relationship between emotional intelligence (EI) and academic stress among tertiary-level students who continued academic activities remotely during the pandemic and the mediating role of social support (SS) in the relationship.

**Methods:**

Using a cross-sectional survey design, 429 students studying business, engineering, social science, and science in Bangladesh provided data via Qualtrics. Using the Structural Equation Modeling in SmartPLS 4 (4.0.8.9), we modeled emotional intelligence as the reflective-formative and social support (support from family, friends, and significant other) and perceived academic stress as the reflective-reflective, second-order constructs. We also conducted a one-way between-groups analysis of variance (ANOVA) to investigate the impact of gender (male and female) and current stage of academic study (Undergraduate year one to four and post-graduation) on emotional intelligence and academic stress, respectively.

**Results and discussion:**

Results show that all the hypothesized relationships are statistically significant: EI is negatively related to perceived academic stress, and SS significantly mediates the relationship between EI and academic stress. Hence, essential strategies are suggested to help students for managing academic stress.

## 1. Introduction

The COVID-19 pandemic has drastically affected the global educational system, including higher education (Martin et al., 2022). Universities worldwide have embraced the online platform, which poses students with unprecedented challenges and ultimately elevates academic stress (Kaspar et al., 2023). Academic stress, though, surfaces from the interaction of multiple factors (García-Martínez et al., 2022); these factors substantially differ between regular and pandemic times. The pandemic caused a transition from traditional face-to-face classes to online classes, (Ullah et al., 2022) bringing new challenges (Crawford et al., 2020) and resulting in academic stress (García-Martínez et al., 2022; Martin et al., 2022).

The pandemic-induced online class has adversely affected students’ psychological wellbeing, characterized by isolation and minimal or no social support (Mosanya, 2020). In addition, students are often anxious about their academic workload and performance while participating in online classes, which adds to the level of stress that they are experiencing (Fitzgerald and Konrad, 2021). Online classes tend to have fewer class interactions and other activities, making students feel like they are falling behind in their learning and causing stress (Hasan and Bao, 2020; Hossain et al., 2021). Therefore, the influence of these challenges of online classes on the psychological health, motivation, and academic performance of students highlights the need for effective strategies to manage academic stress during the pandemic.

In response to online class difficulties caused by the pandemic, students have used emotional intelligence (EI) to manage their academic stress (Chandra, 2021); therefore, it is essential to investigate the potential usefulness of EI in managing academic stress (Merhi et al., 2018). Emotional intelligence has been linked to academic and personal success (Gupta et al., 2023), and an increasing body of research has examined its influence on various aspects of academic life, including stress, anxiety, depression, performance, and deviant behavior (Petrides et al., 2004; Montes-Berges and Augusto, 2007; Nasir and Masrur, 2010; Abdollahi et al., 2013; Aradilla-Herrero et al., 2014; Chacón-Cuberos et al., 2019; Morales and Pérez-Mármol, 2019; Dekker et al., 2020). However, there is limited evidence on the association between EI and academic stress among university students during the COVID-19 pandemic, substantially altering how students learn and communicate (Xie, 2021). In addition, little is known about whether students were cognitively and emotionally ready to learn effectively online (Wang et al., 2022).

Social support, on the other hand, is an essential resource for coping with stress, and it refers to the assistance provided by others in terms of emotional, informational, and tangible support (Ioannou et al., 2019; Zhang et al., 2023). Pandemic-induced online classes have created unique obstacles for students, making social support from family, classmates, and teachers more crucial than ever (Camacho et al., 2021). Emotional support from family has been shown to increase dedication, efficacy, and academic achievement motivation (Song et al., 2015; Budescu and Silverman, 2016), whereas social support from peers has been found to decrease academic stress (Rayle and Chung, 2007; Wilks, 2008). Moreover, teacher social support has been linked to increased academic motivation (Camacho et al., 2021).

Emotional intelligence can positively predict resilience that helps to reduce academic stress among the students (Trigueros et al., 2020). Although several studies identified the impact of emotional intelligence on stress or academic stress, it is important to understand the mechanism through which the relationships of emotional intelligence with resilience and stress have been established. Lopes et al. (2004) identifies that emotional intelligence has a positive impact on quality of interaction of an individual with others such as friends. As people with emotional intelligence are good in maintaining social interactions they are expected to enjoy greater social support (Gallagher and Vella-Brodrick, 2008; Fiorilli et al., 2019). Besides, Lopez-Zafra et al. (2019) observed that students with higher emotional intelligence enjoys higher interaction with others and receives greater social support and this kind of social support helps reducing depression. Furthermore, research indicates that social support assists those with high emotional intelligence to manage academic stress better (Perera and Athanasou, 2012). Individuals with a higher level of emotional intelligence may be better able to seek social support from peers or teachers, enabling them to leverage stressful events. Consequently, social support can mediate between emotional intelligence and academic stress (Kong et al., 2012; Banstola et al., 2020). However, no studies examine the mediating influence of social support on the relationship between emotional intelligence and academic stress, primarily due to online classes during the COVID-19 pandemic. As a result, the current study intends to examine the mediating effect of social support in the association between emotional intelligence and pandemic-induced online academic stress by answering the following research question: ***Does emotional intelligence significantly reduce academic stress caused by online learning during the COVID-19 pandemic, and to what extent does social support mediate the relationship?***

This study uses the JD-R model as its conceptual framework to address the research question. It selects a sample from Bangladesh, where universities suspended face-to-face academic activities in March 2020 (Anwar et al., 2020) and introduced online educational platforms several months later (Al-Amin et al., 2021). Since online learning was unfamiliar to students in Bangladesh (Ullah et al., 2023), the transition to online platforms offered significant challenges, exacerbated by severe resource constraints such as internet connectivity problems, inadequate electricity supply, and a lack of devices and digital skills (Atlam et al., 2022). These challenges emerged as new multipliers of demands on students while physical resources remained scarce, potentially leading to escalating academic stress as postulated under the JD-R model. Furthermore, according to the affective events theory, students may have been more susceptible to stress because of the stressful events that occurred during the pandemic, such as the risk of infection, rising mortality tolls, and lockdowns. Based on the JD-R model, this study, therefore, intends to investigate whether the availability of resources such as EI and social support might alleviate academic stress during this challenging period.

In addition, the influence of demographic factors (gender and current academic year) on the relationship between EI and academic stress during uncertain times remains largely unexplored. Consequently, this study also intends to delve deeper into this issue and investigate the potential impact of these demographic factors on the relationship between EI and academic stress. Specifically, by examining the role of gender and study stage, the study aims to identify potential differences in the relationship between EI and academic stress among various subgroups of university students. The results of this analysis can provide valuable insights into creating focused intervention strategies that can adequately meet the varying requirements of university students, especially in uncertain times. Besides, the overall findings of this study can contribute to the development of effective interventions and support systems to assist students in managing academic stress and achieving academic success on online platforms.

## 2. Literature review and hypotheses

### 2.1. Emotional intelligence and academic stress

Academic stress refers to students’ attitudes and behaviour toward the pressure of accomplishing academic demands and achievements (Karaman et al., 2019). Students’ perceived expectations of academic performance cause academic stress (Ang and Huan, 2006; Deb et al., 2014). Usually, students feel academic pressure to meet the expectation of their parents (Yi et al., 2006; Bedewy and Gabriel, 2015; Deb et al., 2015), academicians (Bedewy and Gabriel, 2015; Jayanthi et al., 2015) and others. So, it is crucial to identify whether the demographic factor of the students (e.g., gender) and their parents’ educational qualifications contribute to academic stress. Deb et al. (2015) concluded that the parent’s educational level and occupation impact their expectations over their children’s academic performance, which causes stress. Although Huan et al. (2006) denied any relationship between gender and academic stress, Misra and McKean (2000) and Jayanthi et al. (2015) reported higher anxiety and academic stress among female students compared to male students and Tangade et al. (2011) reported the opposite, i.e., higher academic stress among male students compared to their counterparts. Besides, the tire of education contributes to academic stress, where students studying in the final year of undergraduate level encounter a higher level of stress (Tangade et al., 2011). Extant literature (Kohn and Frazer, 1986; Misra and Castillo, 2004; Harikiran et al., 2012; Pulido-Martos et al., 2012) also explained that students’ perceived course load and difficulty, test anxiety, final grades, excessive homework, and financial hardship of family cause stress among the students. Khan et al. (2013) explained that severe academic stress and delayed identification hamper students’ ability to study efficiently with proper time management and consequently downgrade students’ academic performance.

Stress has become a widespread phenomenon during the COVID-19 pandemic. COVID-19 resulted in fear, mental stress, anxiety, and depression among university students in Bangladesh (Islam et al., 2020; Sifat, 2021). The pandemic has caused a higher level of mental stress and depression among students (Cao et al., 2020; Liu et al., 2020), which may lead to academic stress as mental health and stress levels impact academic performance (Sano et al., 2015). World Health Organization (WHO) and health experts have warned about the manifestation of these diseases during this situation which may lead to self-harm, chaos in the family, and suicidal cases (Sifat, 2020; WHO, 2020).

The term emotional intelligence has been used occasionally and inconsistent basis before 1990 (Mayer et al., 2011) and in 1990, Salovey and Mayer (1990) defined EI as the subset of social intelligence. The best-selling book ‘Emotional Intelligence’ by Goleman (1995) drew the attention of the term to many researchers (Uzzaman and Karim, 2017), which is an emerging area of study, especially for the educational, medical, and organizational behavior researchers (Chandra, 2021). According to Goleman (1995), some abilities, e.g., recognizing, understanding, and regulating emotions, have a positive relationship with people. In other words, EI is the ability to control interpersonal and intrapersonal emotions to create positive outcomes. Goleman (1995) argued that EI is twice as important as technical skills and more vital than intelligence quotient (IQ) in forecasting positive outcomes; and people should be evaluated not only by their intelligence or professional competence, but also by their own behaviors toward themselves and others.

EI has been found as an important interpreter of students’ activities, such as managing health and academic achievements. Higher levels of emotional intelligence help students to deal with the transition challenges from primary to secondary school (Qualter et al., 2007). Por et al. (2011) highlighted the potential value of EI for nursing students to cope actively and effectively in dealing with stress. Watson and Watson (2016) also indicated an explanatory capacity of emotional intelligence over the academic stress of a sample of American Hispanic students.

Ghosh (2014) concluded a positive correlation between emotional intelligence and academic achievements that might reduce students’ academic stress as academic achievement is negatively correlated with academic stress. Besides, Mohzan et al. (2013) and Pool and Qualter (2012) concluded that emotional intelligence is one of the key determinants of academic success. Iqbal et al. (2021) studied the impact of emotional intelligence on the academic performance of undergraduate students in crisis situation during COVID-19 and found a positive correlation between these two. Valenti et al. (2021) reported that individuals with higher EI are more productive in the workplace and can cope with stress in difficult situations. This study will examine whether such ability to cope with stress through EI is applicable for students to cope with academic stress during a crisis. In light of the discussion, the current study proposes the following hypothesis:

*H1:* Emotional intelligence has a negative relationship with academic stress

### 2.2. Social support as a mediator between emotional intelligence and academic stress

Social support refers to the experience of how an individual is appreciated, loved, and taken care of by the persons present in someone’s life (Gurung, 2006). Deficiency in social support causes depression, loneliness, anxiety, and many psychological problems (Eskin, 2003). Based on the findings of Stroebe and Stroebe (1996) on the impact of social support to reduce stress and other relevant literature, Snyder (1999) argue that emotionally intelligent individuals might access to rich social support network and it would help to reduce their stress. Besides, from previous literature (Yarcheski and Mahon, 1999; Soman et al., 2016; Poots and Cassidy, 2020), the role of social support as a mediator in reducing the adverse impact of stress is well established. Since one’s primary sources of reference are family and friends, support from these sources substantially impacts academic success (Yasin and Dzulkifli, 2010). Through this support, academic stress could be minimized to help them achieve their academic goals. Coffman and Gilligan (2002) concluded that social support largely contributes to stress management and higher life satisfaction among students.

**Figure 1:**
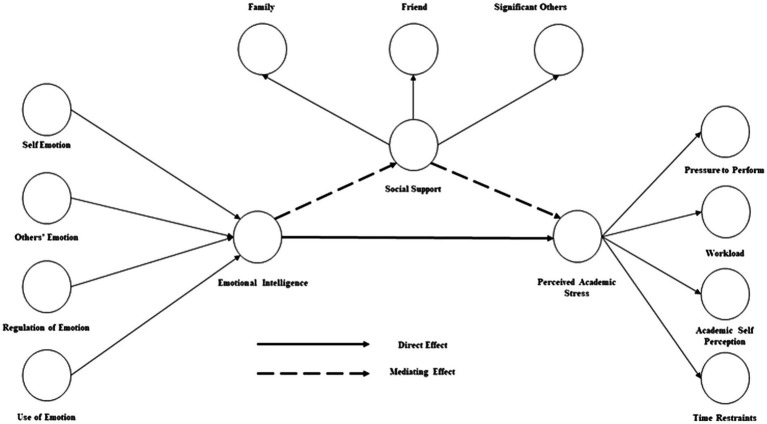
Conceptual model.

Social distancing and isolation created the need for social support during this pandemic, and EI may contribute positively (Zysberg and Zisberg, 2020). Valenti et al. (2021) found a close relationship between EI and occupational stress mediated by social support during COVID-19. Ursin et al. (2020) explained that effective social support helps students to deal with stress. Besides, Rayle and Chung (2007) confirmed that social support helps to deal with academic stress more effectively. Based on the previous studies, the current study offers the following hypothesis (Figure 1):

*H2:* Social support mediates the relationship between emotional intelligence and academic stress

*H2(a):* Friends support mediates the relationship between emotional intelligence and academic stress

*H2(b):* Family support mediates the relationship between emotional intelligence and academic stress

*H2(c):* Significant others support mediates the relationship between emotional intelligence and academic stress

## 3. Methods

### 3.1. Design of the investigation

In this study, a deductive approach is used with a quantitative research design because quantitative research is best for analyzing the relationships between quantitative data and looking into the causes of empirical results (Creswell, 2011; Sekaran and Bougie, 2016). The target respondents of this study are tertiary-level students studying business, engineering, social science, and science in Bangladesh.

The respondent pool was limited to the four public universities in Bangladesh: University of Dhaka, Jahangirnagar University, Rajshahi University, and Chittagong University. These institutions were chosen because they represent the nation’s largest and most prestigious public universities. A public university is a higher education institution funded and regulated by the government. Besides, these institutions hold a great deal of sway in higher education because of their self-regulated status, which gives them more freedom in running their own academic and administrative operations. Furthermore, respondents were only included if they willingly agreed to engage in the study, provided responses in English, and were prepared to spend 10 to 15 min completing the survey.

We chose graduate and undergraduate students in business, engineering, social science, and science as the target respondents for this study because these fields are offered at our chosen university. Besides, these disciplines are prevalent and extensively represented in the country’s higher education institutions. In addition, we selected university-level students because of their degree of maturity and ability to take an active role in the survey process. Furthermore, the recent pandemic has significantly impacted university students, especially those attending public universities.

Through a convenience sampling technique, 500 questionnaire survey forms were given via Qualtrics to the targeted respondents, who were assured that their identities would remain confidential and that their responses would be used solely for research reasons. Besides, they were also notified that their participation is voluntary and that they may opt-out at any moment. While conducting the survey, we followed up with calls and emails after 1 week, 1 month, and 2 months to ensure a prompt response and full collaboration. Despite the attempt, 59 students out of 500 did not return the survey, and 14 responses were eliminated.

Out of the 427 respondents, 57% were male, and the rest were female. 33% of respondents were graduate students, but the majority, 67%, were undergraduate students from various academic levels. Since undergraduate students make up the bulk of the intended audience, most respondents fall within the age range of 21 to 25. In addition, the remaining 12.1 and 11.7% of the respondents fall within the age range of (less than or equal to) 20 years and (more than or equal to) 26 years, respectively. On the other hand, roughly half of the respondents who participated in the survey completed their schooling in the city area, while the remainder were from towns and villages. Most of the respondents (54.3%) live with their parents or other family members (8.2%), while the rest of the students live in dorms (26.6%) and with friends in shared private houses (11%; Table 1).

**Table 1:** Demographic profile of the respondents.

Indicators	Category	Number of respondents	% of respondents
Gender	Male	236	56.9
	Female	191	43.1
Age	> = 20	51	11.7
	21–25	325	76.2
	26 > =	51	12.1
Parents live in	City	215	49.6
	Town	106	23.7
	Village	106	26.7
School location	City	223	50.1
	Town	127	24.9
	Village	77	24.9
Current living status	Dormitories	112	26.6
	With Parents	233	54.3
	With Relatives	35	8.2
	With Friends in Shared Houses	47	11.0
Current level of education	1st Year	100	23%
	2nd Year	51	12%
	3rd Year	48	11%
	4th Year	86	20%
	Undergraduate (total)	285	67%
	Graduate	142	33%

This study employed the G*Power to conduct power analyses specific to model setups. As the model could only have two predictors, this study required 138 participants to test the hypothesis. Besides a sample size of 200 responses or above is appropriate for a sophisticated path model (Kline, 2015). However, we set out to collect data larger than the required number to increase the robustness of the results.

### 3.2. Data analysis

In the current study, the conceptual model was evaluated using SmartPLS 4 (4.0.8.9). PLS-SEM was chosen because CB-SEM is often impractical for estimating complicated models with many latent variables (Hair et al., 2017). A complicated model is characterized by the presence of more than seven variables (Hair et al., 2014). The model used in this current study contains 11 lower-order and three higher-order constructs. Besides, CB-SEM is incapable of running the formative construct. Therefore, PLS-SEM is considered more appropriate for this study.

Typically, PLS models are studied and interpreted in two steps (Hair et al., 2017): the measurement model to assess the reliability and validity, and the structural model to test the stated research hypotheses. In addition, SPSS 26 software was used to perform frequency analysis to acquire descriptive information on the demographic profile.

#### 3.2.1. Normality test

The data distribution is not normal if both the b values for multivariate skewness and kurtosis are higher than 3 and 20, respectively (Kline, 2011). Accordingly, the data distribution of the study is not normal since the Mardia’s multivariate skewness (β = 4.465, *p* < 0.01) and kurtosis (β = 59.263, *p* < 0.01; Table 2).

**Table 2:** Multivariate skewness and kurtosis.

	b	z	*p*-value
Skewness	4.465095	317.021759	*p* < 0.01
Kurtosis	59.263144	11.91325	*p* < 0.01

#### 3.2.2. Common method bias

We ran a thorough collinearity test, and the maximum pathological VIF for all components was 1.299, much below the recommended threshold of 3.3 (Kock and Lynn, 2012). Also, we used the correlation matrix approach (Bagozzi et al., 1991), and Table 3 shows that the highest inter-construct correlation was 0.444, which was below the 0.90 threshold value (Bagozzi et al., 1991). Therefore, these analyses lead to the conclusion that common method bias was not an issue.

**Table 3:** Discriminant validity (HTMT) and correlation matrix.

Constructs	1. SE	2. OE	3. UE	4. RE	5. FMS	6. FRS	7. SOS	8. PP	9. WL	10. ASP	11. TR
1. SE	***1***	***0.292***	***0.370***	***0.420***	***0.283***	***0.224***	***0.187***	***−0.355***	***−0.159***	***−0.200***	***−0.169***
2. OE	0.387	***1***	***0.235***	***0.196***	***0.131***	***0.212***	***0.176***	***−0.252***	***−0.039***	***−0.064***	***−0.009***
3. UE	0.482	0.312	***1***	***0.350***	***0.181***	***0.156***	***0.213***	***−0.505***	***−0.146***	***−0.181***	***−0.002***
4. RE	0.486	0.231	0.424	***1***	***0.287***	***0.212***	***0.214***	***−0.262***	***0.004***	***−0.127***	***−0.146***
5. FMS	0.337	0.167	0.219	0.318	***1***	***0.308***	***0.314***	***−0.241***	***−0.088***	***−0.241***	***−0.122***
6. FRS	0.267	0.261	0.178	0.247	0.357	***1***	***0.347***	***−0.208***	***−0.058***	***−0.121***	***−0.088***
7. SOS	0.220	0.211	0.249	0.231	0.354	0.414	***1***	***−0.225***	***−0.002***	***−0.102***	***−0.086***
8. PP	0.454	0.339	0.652	0.330	0.300	0.245	0.270	***1***	***0.289***	***0.257***	***0.128***
9. WL	0.244	0.064	0.224	0.036	0.127	0.102	0.043	0.436	***1***	***0.406***	***0.245***
10. ASP	0.277	0.123	0.233	0.154	0.287	0.133	0.126	0.367	0.628	***1***	***0.444***
11. TR	0.220	0.044	0.060	0.180	0.151	0.107	0.105	0.193	0.379	0.615	***1***

SE, self emotion; OE, other’s emotion; UE, use of emotion; RE, regulation of emotion; FMS, family support; FRS, friend’s support; SOS, significant others support; PP, pressure to perform; WL, workload; ASP, academic self-perception; TR, time restraints. *Bold and Italic numbers represent latent variables correlation.

### 3.3. Measures

All unobserved constructs used in this study were measured using questions adapted from existing scales (Zimet et al., 1988; Wong and Law, 2002; Bedewy and Gabriel, 2015) and all employed construct items were measured on a Five Point Likert Scale (1 = strongly disagree and 5 = strongly agree; Table 4).

**Table 4:** Measures of the study.

Constructs	References and details	Example items
Emotional intelligence	Wong and Law (2002) 4 dimensions and16 items Reliability ranges from 0.82 to 0.86	“I always know whether or not I am happy” ***(Dimension: Self-Emotional Appraisal)*** “I am a good observer of others’ emotions” ***(Dimension: Others’ Emotional Appraisal)*** “I am a self-motivated person.” ***(Dimension: Use of Emotion)*** “I have good control of my own emotions.” ***(Dimension: Regulation of Emotion)***
Perceived academic stress	Bedewy and Gabriel (2015) 18 items Scale Reliability 0.70	“The unrealistic expectations of my parents stress me out” ***(Dimension: Pressure to Perform)*** “The size of the curriculum (workload) is excessive” ***(Dimension: Workload)*** “I am confident that I will be successful in my future career” ***(Dimension: Academic Self-Perception)*** “The time allocated to classes and academic work is enough” ***(Dimension: Time restraints)***
Perceived social support	Zimet et al. (1988) 3 dimensions and 12 items Scale Reliability 0.88	“I can talk about my problems with my family” ***(Dimension: Family Support)*** “I can count on my friends when things go wrong” ***(Dimension: Friends Support)*** “There is a special person who is around when I am in need” ***(Dimension: Significant Others Support)***

## 4. Results

### 4.1. Assessment of measurement model

Hair et al. (2017) advised demonstrating convergent and construct validity when evaluating a measuring model. To establish convergent validity, we utilized the loading of each item on its associated variable, average variance extracted (AVE), and composite reliability (CR; Hair et al., 2014).

Table 5 shows that all item loadings exceeded the recommended value of 0.7. However, the items self-emotion 4 (0.564), regulation of emotion 3 (0.645), pressure to perform 4 (0.524), workload 3 (0.673) and time restraints 3 (0.645) were retained due to the acceptable values for AVE and CR (Hair et al., 2014). We found CR values above 0.70 (Chin, 2010) to be indicative of construct validity (Table 1). Table 5 shows that the AVE values for each latent variable in the measurement model surpassed the required value of 0.5 (Hair et al., 2017).

**Table 5:** Lower order reliability and convergent validity.

Constructs	Items	Loadings	AVE
1. Self-emotion	SE1	0.813	0.584
CR = 0.846	SE2	0.84	
	SE3	0.808	
	SE4	0.561	
2. Others’ emotion	OE1	0.791	0.645
CR = 0.845	OE2	0.81	
	OE4	0.809	
3. Use of emotion	UE1	0.728	0.579
CR = 0.846	UE2	0.716	
	UE3	0.781	
	UE4	0.814	
4. Regulation of emotion	RE1	0.843	0.654
CR = 0.882	RE2	0.862	
	RE3	0.643	
	RE4	0.865	
5. Family support	FMS1	0.835	0.714
CR = 0.909	FMS2	0.9	
	FMS3	0.806	
	FMS4	0.837	
6. Friend’s support	FRS1	0.85	0.646
CR = 0.881	FRS2	0.759	
	FRS3	0.798	
	FRS4	0.807	
7. Significant other support	SOS1	0.871	0.764
CR = 0.928	SOS2	0.876	
	SOS3	0.903	
	SOS4	0.846	
8. Pressure to perform	PP1	0.86	0.58
CR = 0.842	PP2	0.88	
	PP3	0.728	
	PP4	0.524	
9. Workload	WL1	0.871	0.686
CR = 0. 813	WL2	0.783	
10. Academic self perception	ASP 1	0.774	0.552
CR = 0.831	ASP 2	0.777	
	ASP 3	0.735	
	ASP13	0.682	
11. Time restraints	TR 1	0.875	0.781
CR = 0.877	TR 2	0.892	

Besides, to test the discriminant validity, we used the Heterotrait–Monotrait ratio of correlations (HTMT; Zailani et al., 2019). The HTMT ratio should be lower than 0.85 to verify discriminant validity (Kline, 2016). Table 3 demonstrates that all values passed the HTMT ≤0.85 threshold, validating the measurement model’s discriminant validity.

In addition, we modeled emotional intelligence as the reflective-formative, and social support and perceived academic stress as the reflective-reflective, second-order constructs. Emotional intelligence is established through self-emotion, others’ emotions, significant others’ emotions, and emotion regulation. Since emotional intelligence is the ability to control one’s emotions to express them effectively and responsibly, all the dimensions are equally important to measure one’s EI. Therefore, it is not possible to quantify EI by omitting any lower-order constructs. In addition, emerging research on emotional intelligence considers EI as a formative rather than a reflective construct (Gentina et al., 2018; Iqbal et al., 2021).

Likewise, we modeled social support and perceived academic stress as reflective-reflective second-order constructs. Family support, friends’ support, and significant others’ support are the dimensions of social support, while pressure to perform, workload, academic self-perception, and time restraints are the dimensions of perceived academic stress. We used second order because it reduces the number of relationships (and thus the number of hypotheses to be tested) in the structural model, making the PLS Path model more compact and easier to comprehend (Hair et al., 2014). We employed a two-stage procedure to generate these second-order constructs (Becker et al., 2012), which included computing the latent variable scores and using these values as the items for the second-order constructs. To create these second-order constructs, we used a two-stage approach (Becker et al., 2012), which involves calculating the latent variable scores and using these scores as the items for the second-order constructs. The items used to measure this study’s two second-order reflective-reflective constructs are valid and reliable, as shown in Table 6.

**Table 6:** Second order reliability and validity.

Second order construct	Dimension	Weights/Loadings	*p*-value/CR	VIF/AVE
Emotional intelligence	Self-emotions	0.438	0.000	1.357
(Type: Formative)	Others’ emotion	0.221	0.008	1.119
	Use of emotion	0.441	0.000	1.245
	Regulation of emotion	0.301	0.000	1.286
Social support	Family support	0.770	CR = 0.783	AVE = 0.547
(Type: Reflective)	Friend support	0.720		
	Significant other support	0.730		
Perceived academic stress	Pressure to perform	0.875	CR = 0.762	AVE = 0.453
(Type: Reflective)	Workload	0.641		
	Academic self perception	0.684		
	Time restraints	0.489		
Second order discriminant validity		Perceived academic support		
	Social support	HTMT = 0.437		
	Family support	HTMT = 0.319		
	Friend’s support	HTMT = 0.218		
	Significant other support	HTMT = 0.192		

Emotional intelligence, on the contrary, was modeled as Type II second-order measurement (reflective-formative); the accuracy of this formative measurement was assessed by looking at the weights, t-values, and the variance inflation factor (VIF). Table 6 displays the assessment for formative measurement.

### 4.2. Assessment of structural model

**Figure 2:**
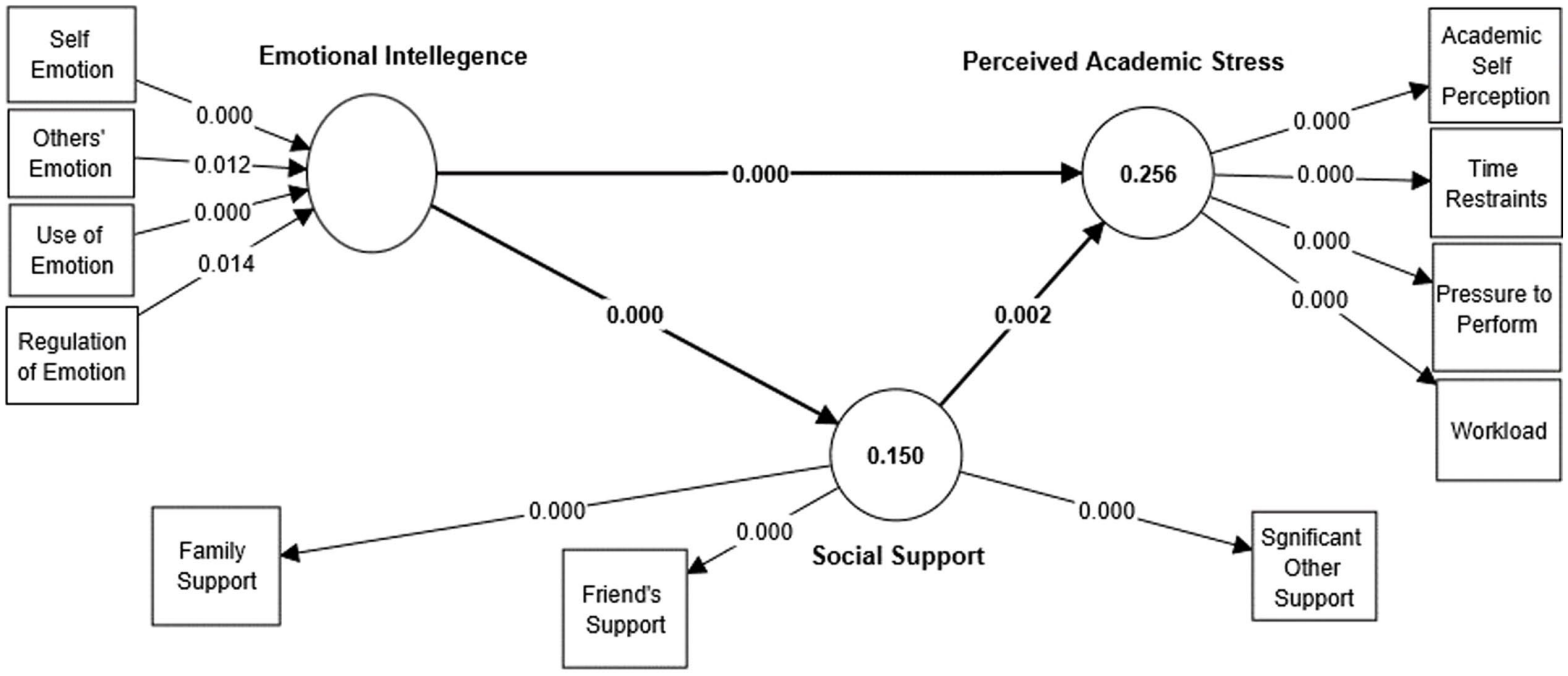
Structural model (a).

**Figure 3:**
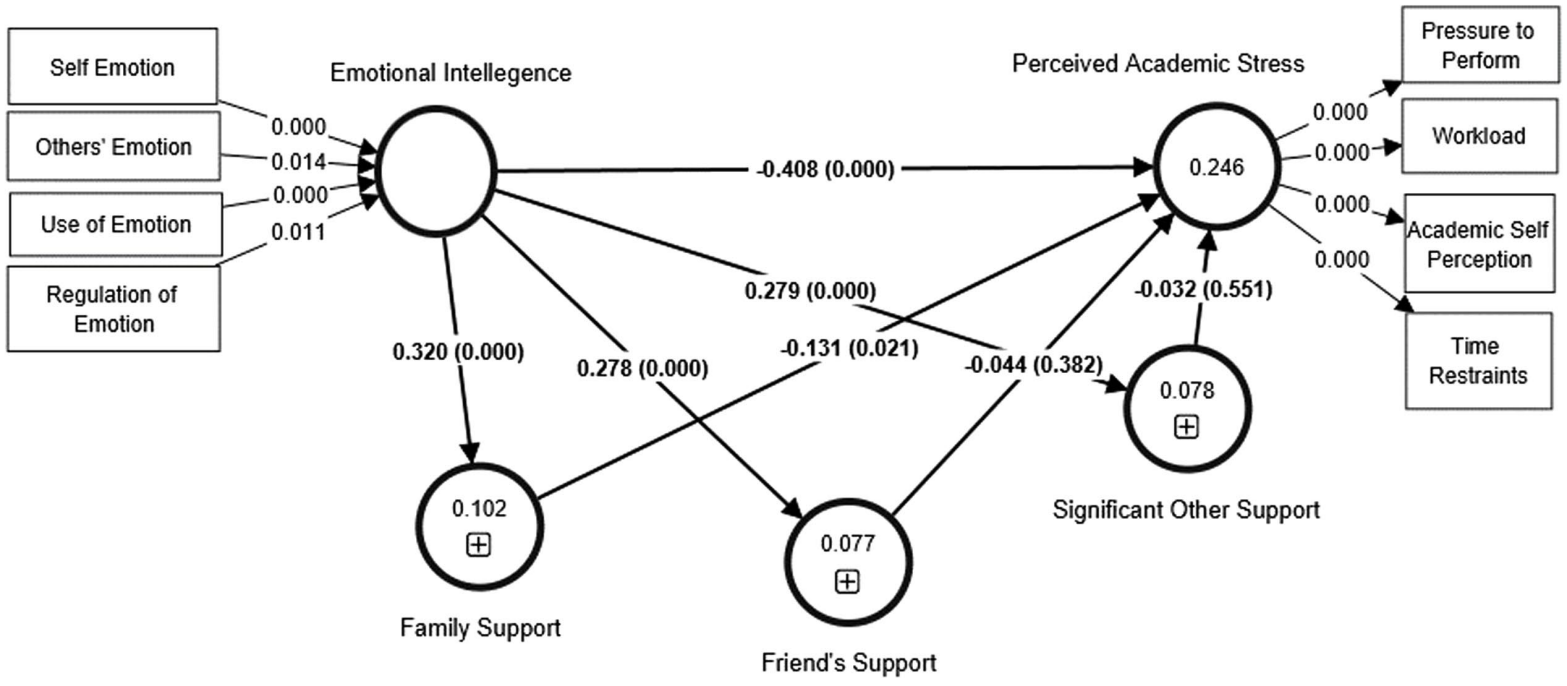
Structural model (b).

Sarstedt et al. (2021) indicated that the path coefficients and their accompanying t-values were derived using 5,000 bootstrap subsamples to test the proposed study hypotheses. Table 7 and Figures 2, 3 demonstrate that EI has a statistically significant negative association with PAS (β = −0.408, t = 7.327, *p* < 0.05). Therefore, H1 is supported. Likewise, it is evident from Table 7 and Figure 2 that EI has a significant indirect effect on PAS by social support (SS; β = −0.060, t = 2.661, *p* < 0.05). Besides, the direct effect of EI on SS (β = 0.389, t = 8.046, *p* < 0.05) and SS on PAS (β = −0.152, t = 2.951, *p* < 0.05) are also significant. Thus, H2 is supported, and it can be said that SS mediates the relationship between EI and PAS, and the mediation is partial mediation.

**Table 7:** Test of hypotheses.

Hypothesis	Path relationship	β	Std. Deviation	*t*- value	*p*-value	5.00%	95.00%	Decision	f square
1	EI -> PAS	−0.408	0.056	7.327	0.000	−0.489	−0.304	Supported	0.186
2	EI -> SS -> PAS	−0.060	0.023	2.661	0.004	−0.098	−0.025	Supported	
2(a)	EI -> FRS -> PAS	−0.012	0.015	0.803	0.211	−0.037	0.013	Not Supported	
2(b)	EI -> FMS -> PAS	−0.042	0.019	2.162	0.015	−0.074	−0.011	Supported	
2 c)	EI -> SOS -> PAS	−0.009	0.016	0.568	0.285	−0.036	0.016	Not Supported	
		R Square	0.256 (PAS)	0.150 (SS)	Q Square	0.203 (PAS)	0.135 (SS) SRMR = 0.086		

EI, emotional intelligence; SS, social support; FMS, family support; FRS, friend’s support; SOS, significant others support; PAS, perceived academic stress.

Moreover, the study delves deeper and examines the mediating effect of family, friends and significant other support in the linkage between EI-PAS under social support. Table 7 and Figure 3 highlight that the indirect effect of EI on PAS mediated by friend’s support (FRS; β = −0.012, *t* = 0.803, *p* > 0.05, CI has value 0 included) and significant others support (SOS; β = −0.009, *t* = 0.568, *p* > 0.05, CI has value 0 included) is not statistically significant. So, FRS and SOS do not mediate the EI-PAS linkage. Thus H2(a) and H2(c) are not supported. However, Table 7 and Figure 3 highlight that the indirect effect of EI on PAS mediated by family support (FMS) is statistically significant (β = −0.012, *t* = 0.803, *p* > 0.05). Besides, the direct effect of EI on FMS (β = 0.320, *p* < 0.05) and FMS on PAS (β = −0.131, *p* < 0.05) are also significant. Thus, H2 (b) is supported, and it can be said that FMS mediates the relationship between EI and PAS, and the mediation is also partial mediation.

In the current study, the $R^{2}\;$ values, a measure of the accuracy of the model’s predictions, were 0.250 and 0.151 for perceived academic stress and social support, respectively. In addition, the Stone-Geisser $Q^{2}\;$ (cross-validated redundancy) value was computed to quantify the predictive relevance based on a blindfolding procedure in PLS (Teo et al., 2017; Saghapour et al., 2018). According to Chin (2010), the model exhibits predictive relevance if the value of $Q^{2}\;$ is greater than 0. The results show that the $Q^{2}\;$ values for perceived academic stress (0.210) and social support (0.136) are more than 0, verifying the predictive relevance of the endogenous variables in this study.

In structural models, on the other hand, the effect size is regarded as one of the most crucial indicators of statistical power. Therefore, Cohen (1988) established the thresholds for measuring the influence of effect size $f^{2}$ at the structural level: 0.02 for a small, 0.15 for a medium, and 0.35 for a large effect. Table 7 revealed that for $f^{2}$ emotional intelligence has a moderate effect on both perceived academic stress and social support, while social support has a small effect on perceived academic stress. Moreover, we evaluated the structural model’s collinearity. Results indicate that the VIF values of all predictor variables are below the cautious threshold of 5 (1.111 to 1.591), indicating the absence of multicollinearity problems (Hair et al., 2017).

### 4.3. Analysis of variance

A one-way between-groups analysis of variance (ANOVA) was performed to investigate the impact of gender (male and female) and current tier of academic study (1st year, 2nd year, 3rd year, 4th year, and Master) on emotional intelligence and perceived academic stress, respectively. Investigation of the skewness, kurtosis, and Shapiro–Wilk statistics showed that the assumption of normality for the dependent variable (emotional intelligence and perceived academic stress) was not violated. Besides, Levene’s statistics were not significant for both EI [*F* (1,424) = 1.788, *p* = 0.0.182] and PAS [*F* (4,421) = 2.114, *p* = 0.078]; hence the assumption of homogeneity variance can be made.

There was a statistically significant difference in EI score at the p < 0.05 level for the two groups: F (1,424) = 7.893, *p* = 0.002. Therefore, it is comprehended that male students have higher EI than females, as evident from this current study. The effect size, calculated using eta squared, was 0.018. Based on Cohen’s criteria, the effect size appears to be small. Therefore, despite the result showing that males and females have different emotional intelligence, the difference is negligible and limited to the particular context.

Likewise, there was a statistically significant difference at the *p* < 0.05 level in perceived academic stress score for five groups: F (4,421) = 5.374, *p* = 0.000. The effect size, calculated eta squared, was 0.048. Post-hoc comparisons using the Tukey HSD test revealed that the degree of academic stress in each year of schooling is very different. Student stress levels vary widely from first year to final year, third year to final year, and final year to post-graduation. Among the five groups, master’s students (M = 2.970, SD = 0.570) have fewer level of stress, whereas final-year students (M = 3.970, SD = 0.689) have higher stress levels. In particular, the level of perceived academic stress is significantly different between the second-year (M = 3.329, SD = 0.559) and master’s students, as well as final-year students and masters’ students. However, inter-year levels of perceived academic stress among the first-year (M = 3.049, SD = 0.509), third-year (M = 3.052, SD = 0.618) and master’s students did not differ significantly.

## 5. Discussion and conclusion

### 5.1. Discussion of findings

Despite plenty of research in the field of emotional intelligence, academic stress, and social support, there is limited evidence on the association between EI and academic stress among university students during the COVID-19 pandemic. Besides, no prior studies examine the mediating influence of social support on the relationship between emotional intelligence and academic stress, especially due to online classes during the COVID-19 pandemic. Therefore, this study analyzes the relationship between emotional intelligence and perceived academic stress and the mediation effect of social support (family, friends, and significant others) in the relationship between emotional intelligence and academic stress of tertiary-level students when they continue academic activities during a crisis like the COVID-19 pandemic.

In the first hypothesis, emotional intelligence is assumed to have a negative association with academic stress. The result validates the hypothesis that students with greater EI experience less stress (Por et al., 2011; Valenti et al., 2021). EI is a personal resource that helps to combat negative situations positively (Gupta and Kumar, 2010). Valenti et al. (2021) also reported a similar result on occupational stress and EI during a challenging time, and MacCann et al. (2020) underscored that EI is a vital tool to cope with students’ stress and negative emotions. Emotional intelligence is thus a protective personal resource for students navigating academic stress, as it enables them to effectively manage and comprehend their emotions, nurturing positive relationships with others while coping with negative emotions.

The second hypothesis hypothesized that social support mediates the negative association between emotional intelligence and academic stress caused by online classes. The hypothesis was tested, and the results revealed that social support mediates the connection between EI and PAS due to online classes. Though academic stress in the context of pandemic-induced online classes has not yet been investigated, prior research has examined the mediating effect of social support in the EI and perceived stress (Ursin et al., 2020; Zysberg and Zisberg, 2020; Valenti et al., 2021) and found that social support mediates the EI and perceived stress linkage. Similarly, this study’s results suggest that students can rely on social support to help them cope with academic stress in the face of unforeseen circumstances.

Students’ communities are more susceptible to stress and trauma than working professionals because of the understanding of subjective well-being of life and EI increase with age (Chen et al., 2016). While pursuing tertiary education, they mostly migrate from rural to urban areas and stay away from family members, making their life more stressful. Under these circumstances, social support through frequent interactions, positive feedback, and counselling can drive down stress and improve mental health. They may also develop emotional intelligence through this process because they might better regulate disruptive emotions.

Under social support in the second hypothesis, the study also investigates the specific mediating effect of familial support, friend support, and significant other support on the relationship between EI and PAS. The results indicate that family support significantly mediates the relationship between emotional intelligence and perceived academic stress in the context of online classes. However, it was found that the mediating effects of friend support and significant other support were not statistically significant. These findings are relevant for students in public universities in Bangladesh, many of whom encounter economic challenges and lack access to necessary resources for online learning. The pandemic exacerbated these difficulties, as students struggled with limited access to smartphones, personal computers, and related equipment. The burden imposed on students was alleviated to a greater extent when they received support from their families, who played a crucial role in providing necessary resources and fostering an emotionally stable environment. In contrast, the limited social detachment caused by the pandemic led to decreased access to friends and others, resulting in insignificant mediating effects of friend and significant other support. These findings highlight the pivotal role of family support in mitigating academic stress and underscore the impact of economic constraints on students’ online learning experiences.

In addition to the hypotheses, the study provides some insightful findings. It is improbable for all students to possess an equivalent level of EI, leading them to react distinctively in response to stressful situations encountered throughout their academic journey and challenging periods. Therefore, the study examined levels of EI between male and female students. The result reveals that male students possess a significantly higher EI than female students, indicating the higher vulnerability of female students during a crisis that demands more empathetic interactions. However, the effect size of the difference of EI between the male and female is very small (η $p^{2}$ = 0.018), implying that the generalizability of the findings is limited within the Bangladeshi context.

Likewise, the study also examined the degree of academic stress in each year of schooling at the tertiary level. The result reveals that student stress levels vary widely from first year to final year, third year to final year, and final year to post-graduation. The effect size of the differences is moderate (η $p^{2}$ = 0.048), which implies substantial disparities between student years in school and their perspectives on academic stress.

### 5.2. Implications

In a world of growing stress arising out of many academic, environmental, socio-cultural, and psychological factors (Chandra, 2021), it is essential to promote the students’ EI. Promoting skills related to emotion management of students will enable them to achieve better academic results (MacCann et al., 2011). Since stress grows from uncontrolled factors, actions are needed to harness EI skills. EI training to be included in tertiary education curriculum that can help students combat growing stress levels in their academic life. Since EI is more of learning skills than inherited, training can boost EI. Higher education institutions can also practice assessing students’ EI and stress levels to design coping mechanisms for different groups of students. This will result in better stress management at the institutional level, protecting vulnerable students from distress and a tendency to commit suicide. Institutions can also convey the assessment results to the concerned family members to take corrective measures for handling those with higher levels of stress or lower levels of EI.

Apart from teaching EI skills, higher education institutions also need to promote an environment of empathy and emotional support for students by providing them with access to mental health services, encouraging open dialogue about their emotional experiences, and educating faculty and staff teams about the need for emotional intelligence and interactions with positive and encouraging language inside and outside the classroom. Since COVID-19 can negatively affect a person’s emotional and psychological wellbeing, institutions should prioritize providing students with counseling and other mental health services. Institutions may also consider incorporating mindfulness practices and other stress-reduction techniques into the curriculum to promote emotional health and resilience. Although EI instruction might help students deal with the pressures of institutions, it is vital to remember that stress is a complicated issue with many potential causes. Therefore, institutions must adopt a comprehensive approach to address emotional, environmental, social, and psychological factors contributing to stress. Besides, systemic concerns, such as high academic expectations, financial stress, and limited access to resources, can also be addressed by institutions. Therefore, adjustments in policy, such as reducing workload or increasing support for disadvantaged students, as well as community outreach and advocacy efforts, are necessary to raise awareness of the issue. Besides, to design better architecture for handling students’ academic stress, university administrators need to facilitate institutional-level integration with subjects like clinical psychology. Furthermore, family members, friends, and significant people from society, like teachers, relatives, and role models, can emotionally connect with students, assist students in developing their EI, and reduce academic stress.

### 5.3. Limitations and future research directions

This study’s findings are important and well-supported, but the cross-sectional survey design limits our ability to conclude the temporal relationships among participants’ emotional intelligence, social support, and perceived academic stress. Besides, participants in the study are restricted to Bangladesh, so the results may not be generalizable to other economic, cultural, and geographical contexts. Finally, this study used self-report measures, which could have introduced response or social desirability bias into the study.

Henceforth, research in the future could use longitudinal or experimental designs to investigate the links between EI, social support, and academic stress. Possible areas for future research also include how cultural and environmental factors, such as differences in social norms, educational systems, and job opportunities, affect the association between emotional intelligence, social support, and academic stress. Other than perceived academic stress, academic outcomes like performance and motivation may also be influenced by emotional intelligence, which might be explored in future studies. Furthermore, future research could investigate the potential mediators and moderators (e.g., coping strategies or personality traits) in the linkage between emotional intelligence and academic stress. As the current study reported that gender and academic seniority cause a significant difference in stress levels, future studies can address the potential sources of stress for each group and their coping strategies.

## Data availability statement

The raw data supporting the conclusions of this article will be made available by the authors, without undue reservation.

## Ethics statement

Ethical review and approval was not required for the study on human participants in accordance with the local legislation and institutional requirements. Written informed consent from the [patients/ participants OR patients/participants legal guardian/next of kin] was not required to participate in this study in accordance with the national legislation and the institutional requirements.

## Author contributions

MU: conceptualization of Idea, writing of paper (introduction, discussion and implication) and overall coordination. SA: data collection, writing of paper (Introduction, literature review), and editing. MA: Conceptualization of idea, data collection, and cleaning, writing of the paper (introduction, literature review, measures). MI: data cleaning and analysis, writing of paper (introduction, methods, results and conclusion). All authors contributed to the article and approved the submitted version.

## Conflict of interest

The authors declare that the research was conducted in the absence of any commercial or financial relationships that could be construed as a potential conflict of interest.

## Publisher’s note

All claims expressed in this article are solely those of the authors and do not necessarily represent those of their affiliated organizations, or those of the publisher, the editors and the reviewers. Any product that may be evaluated in this article, or claim that may be made by its manufacturer, is not guaranteed or endorsed by the publisher.
